# Metal–Organic Framework Derived Ni_2_P/FeP@NPC Heterojunction as Stability Bifunctional Electrocatalysts for Large Current Density Water Splitting

**DOI:** 10.3390/molecules28052280

**Published:** 2023-02-28

**Authors:** Huimin Jiang, Shuo Zhang, Qiuju Fu, Liting Yan, Jun Zhang, Xuebo Zhao

**Affiliations:** 1 State Key Laboratory of Heavy Oil Processing, College of Chemistry and Chemical Engineering, China University of Petroleum (East China), Qingdao 266580, China; 2 School of Materials Science and Engineering, Qilu University of Technology (Shandong Academy of Sciences), Jinan 250353, China

**Keywords:** metal-organic frameworks, heterojunction, electrocatalysis, large current density, water splitting

## Abstract

The construction of heterojunction has been widely accepted as a prospective strategy for the exploration of non-precious metal-based catalysts that possess high-performance to achieve electrochemical water splitting. Herein, we design and prepare a metal-organic framework derived N, P-doped-carbon-encapsulated Ni_2_P/FeP nanorod with heterojunction (Ni_2_P/FeP@NPC) for accelerating the water splitting and working stably at industrially relevant high current densities. Electrochemical results confirmed that Ni_2_P/FeP@NPC could both accelerate the hydrogen and oxygen evolution reactions. It could substantially expedite the overall water splitting (1.94 V for 100 mA cm^−2^) which is close to the performance of RuO_2_ and the Pt/C couple (1.92 V for 100 mA cm^−2^). In particular, the durability test exhibited that Ni_2_P/FeP@NPC delivers 500 mA cm^−2^ without decay after 200 h, demonstrating the great potential for large-scale applications. Furthermore, the density functional theory simulations demonstrated that the heterojunction interface could give rise to the redistribution of electrons, which could not only optimize the adsorption energy of H-containing intermediates to achieve the optimal Δ*G*_H_* in a hydrogen evolution reaction, but also reduce the Δ*G* value in the rate-determining step of an oxygen evolution reaction, thus improving the HER/OER performance.

## 1. Introduction

Electrocatalytic overall water splitting exhibits great potential in sustainable hydrogen production, which is composed of two half reactions, one being a hydrogen evolution reaction (HER) and the other being an oxygen evolution reaction (OER) [1,2]. However, the efficiency of water splitting has been strongly hindered by the sluggish kinetics process [3]. Previous reports have proved that the traditional noble metal–based electrocatalysts have high catalytic performance on HER or OER, such as iridium and ruthenium oxides for OER and platinum for HER [4,5]. However, these catalysts might only be suitable for the half reaction, and commonly suffer from expensive costs and scarce reserves. In general, several requirements must be met for water splitting catalysts to achieve practical applications: (1) stable and high active sites at large current density [6]; (2) fast electron transfer [7]; and (3) low-cost and easy access [8]. Recently, some progress was achieved in electrochemical water splitting; nevertheless, most of the reported electrodes were evaluated in terms of overpotentials and stability at low current densities, which are not practical for large-scale applications [9]. It remains an urgent need to explore high-performance catalysts with large current density and robust durability for overall water splitting [3,10]. The development of non-noble metal bifunctional electrocatalysts with robust and stable catalytic performance has exhibited great potential in practical applications, and the exploration of bifunctional catalysts with both HER and OER will significantly simplify the water splitting devices.

Currently, tremendous efforts have been made in developing efficient and scalable overall water splitting electrocatalysts [11,12]. It has been established that the transition metal phosphides, such as Fe and Ni-based phosphides, possess superior electrical conductivity and high electron density near the Fermi level. They can effectively improve the intrinsic conductivity of materials to realize large charge carrier transfer efficiency and catalytic capability in the electrocatalytic process [13,14]. The positively charged metal cations in metal phosphides can be regarded as the hydroxyl receptors, and negatively charged phosphorous active sites can accelerate the dissociation of H_2_ to boost HER activity. Furthermore, the introduction of secondary metal into the metal center can increase the active sites, optimize the *e*_g_ orbitals, change the charge transfer path, and modulate the electronic structure for better electrocatalytic capabilities in overall water splitting [15]. To date, however, the uncontrolled generation of multiple segregated phases due to the reactive difference of metal centers still makes it challenging to synthesize homogeneous bimetallic phosphides.

As a new class of crystalline materials, metal-organic frameworks (MOFs) are constructed with metal nodes and organic ligands [16,17,18]. Their compositions and chemical environments can be easily modulated and systematically designed via changes of metal ions in the MOF precursor, or by annealing processes in various environments [19]. In addition, the structure and morphology of the obtained products can be well preserved [20,21]. For now, numerous different functional MOFs have been created by changing the metal ions/clusters and organic linkers which have been regarded as promising candidates for carbon-based materials [22,23].

In this work, we report the designing and fabricating of Ni_2_P/FeP heterojunction encapsulated in N, P-doped carbon frameworks (donated as Ni_2_P/FeP@NPC) through a bimetallic MOF precursor topochemical conversion, aiming at boosting both the activity and durability of OER and HER electrocatalysts. First, we synthesized the Ni/Fe MOF, which was then followed by pyrolysis, and the phosphating of the Ni_2_P/FeP@NPC was conducted. Because of the remarkable HER and OER activity, this bifunctional Ni_2_P/FeP@NPC was integrated in an alkaline electrolyzer as both the anode and cathode electrodes. The experimental results demonstrated that only a cell voltage of 1.64 V could deliver 10 mA cm^−2^, and a cell voltage of 2.37 V was needed to deliver 500 mA cm^−2^ with 200 h durability, outperforming most catalysts with similar functions and those of precious metal catalysts (Pt/C//RuO_2_, 2.41 V for 500 mA cm^−2^).

## 2. Results and Discussion

**Figure 1:**
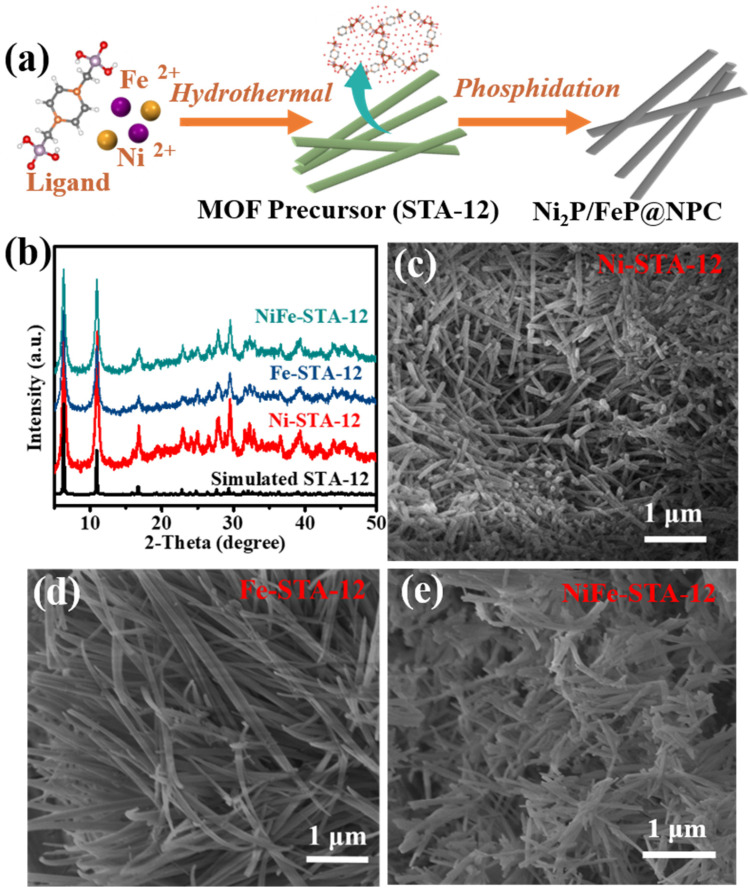
(**a**) Synthetic routes in the schematic illustration of the MOF precursor and the derived Ni_2_P/FeP@NPC; (**b**) XRD patterns of the obtained MOFs; (**c**–**e**) are the SEM images of Ni-STA-12, Fe-STA-12, and NiFe-STA-12, respectively.

The synthetic step of the Ni_2_P/FeP@NPC catalysts is illustrated in Figure 1a. The MOFs precursors (denoted as STA-12) were prepared with a hydrothermal method according to previous work with small modifications (all details can be obtained from the Supporting information) [24]. The metal nodes in STA-12 were tailored by adjusting the species of metal salts in the preparation process. As shown in Figure 1b, the powder X-ray diffraction (PXRD) patterns demonstrated that all STA-12 crystals possessed the same PXRD pattern and exhibited high agreement with the reference [24], which confirmed that the bimetallic STA-12 crystals possessed the same crystal structure with single-metal-center STA-12. The scanning electron microscopy images (SEM) in Figure 1c–e show that all the STA-12 exhibited similar regular nanorod morphologies. Interestingly, as shown in Figure 1e, compared with single-metal-center STA-12, the bimetallic STA-12-FeNi exhibited much slender morphologies, which might be caused by the presence of Fe that caused the MOF crystal growing along specific lattice plane, limiting the growth in other lattice planes.

**Figure 2:**
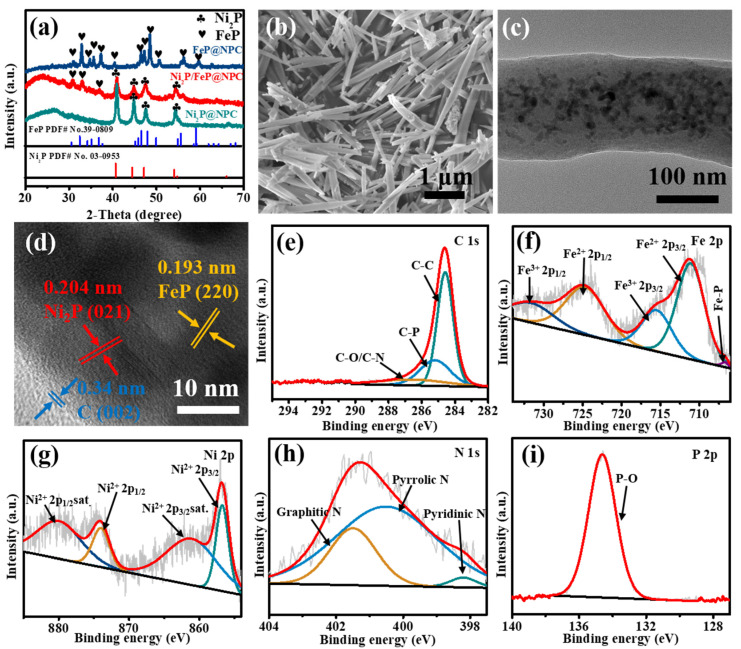
(**a**) XRD pattern of Ni_2_P@NPC, Ni_2_P/FeP@NPC and FeP@NPC; (**b**) SEM images of Ni_2_P/FeP@NPC; (**c**) TEM images of Ni_2_P/FeP@NPC; (**d**) HRTEM image of Ni_2_P/FeP@NPC; High-resolution XPS spectra of (**e**) C, (**f**) Fe (**g**) Ni, (**h**) N and (**i**) P in Ni_2_P/FeP@NPC.

After carbonization and phosphorization of the MOF precursor, the XRD patterns of Ni_2_P@NPC, FeP@NPC, and Ni_2_P/FeP@NPC are shown in Figure 2a. The peaks at 2θ of 31.83°, 35.33°, 40.75°, 44.64°, 54.15° and 54.37° are matched well with hexagonal Ni_2_P crystal planes (JCPDS No. 65-3544) of (011), (111), (021), (210), (300) and (002), respectively. Meanwhile, the peaks of FeP at 2θ of 32.78°, 37.18°, 46.32°, 46.98°, 48.36°, 56.09° are also illustrated, which belong to the (011), (111), (112), (202), (211) and (212) crystal planes respectively, and exhibited high agreement with orthorhombic FeP (JCPDS No. 65-2595). As shown in Figure S1, the obtained Raman spectrums of the all catalysts exhibited two broad and obvious peaks positioned at 1355 and 1603 cm^−1^ corresponding to the typical D and G bands of graphene, respectively. The detection of these two peaks demonstrated the existence of substantial defects or disordered sites, which might be due to the concurrent doping and absence of C atoms. The SEM images were then collected to explore the morphology of the materials after the carbonization of MOF precursors (Figure 2b and S2). It can be seen that the morphology of Ni_2_P@NPC, Ni_2_P/FeP@NPC and FeP@NPC are similar to the MOF precursors, indicating that the morphology can be well preserved during the phase transformation process. As shown in Figure 2c, the transmission electron microscopy (TEM) image exhibited that Ni_2_P/FeP@NPC possessed a typical rod-shaped morphology with a diameter of approximately 120 nm. From high-resolution TEM (HRTEM) images (Figure 2d), lattice fringes could be observed in the Ni_2_P/FeP@NPC, in which the spacing of 0.204 nm was attributed to the (021) planes of Ni_2_P. In contrast, the lattice fringe with an inter-planar distance of 0.193 nm was corresponding to the (220) crystal planes of FeP. All of these results exhibited high consistency with the XRD analysis, proving the successful preparation of Ni_2_P/FeP@NPC. As shown in Figure 2d, the obvious phase boundary between the marked Ni_2_P and FeP proved the production of a heterostructure, which could generate an interfacial bonding effect that facilitates the exposure of more active sites. Furthermore, the lattice fringe with a spacing distance of 0.34 nm could be ascribed to the (002) plane of graphitic carbon, confirming the existence of carbon substrate through the pyrolysis process. The energy-dispersive X-ray spectrometry (EDX) mapping was applied to reveal the element distribution of Ni_2_P/FeP. As shown in Figure S3, the elements of C, Ni, Fe, P, and N are homogeneously dispersed in the structures, demonstrating the successful preparation of Ni_2_P/FeP@NPC compounds.

X-ray photoelectron spectroscopy (XPS) was employed to evaluate the valence state and composition of the prepared samples more accurately. The XPS survey scan spectrum in Figure S4 indicated the existence of C, Fe, Ni, N, and P in the architecture. The high-resolution spectra of these elements further confirmed the formation of Ni_2_P/FeP@NPC. The C 1s XPS spectrum in Figure 2e could be deconvoluted into three peaks positioned at 286.5, 285.2, and 284.6 eV, which were ascribed to the C-O/C-N, C-P, and C-C/C = C groups, respectively [25]. The existence of C-O, C-P, and C-N offered numerous anchoring sites for electrochemically active materials to inhibit agglomeration or disengagement of particles from electrodes. As shown in Figure 2f, two main spin-orbit doublets in the Fe 2p spectrum could be divided into four components: the first doublet located at 711.1 eV and 724.6 eV belonged to Fe^2+^ 2p_3/2_ and Fe^2+^ 2p_1/2_, and the second doublet located at 715.5 eV and 731.6 eV was ascribed to the splitting peaks of Fe^3+^ 2p_3/2_ and Fe^3+^ 2p_1/2_, respectively. In addition, the small peak positioned at 706.7 eV indicated the existence of metallic Fe [26]. As shown in Figure 2g, the two spin-orbit doublet peaks at 856.7 and 874.0 eV with two shake-up satellites at 861.1 and 880.0 eV indicated the existence of oxidized Ni species in Ni_2_P/FeP@NPC, ascribed to the surface oxidation in air [27]. As shown in Figure 2h, the peak located at 133.7 eV in the P 2p region was attributed to the oxidized phosphide (P-O) [26]. Furthermore, the N 1s spectrum in Figure 2i could be deconvoluted into pyridinic-N (398.2 eV), pyrrolic-N (400.5 eV), and quaternary-N (401.5 eV), confirming the incorporation of N into Ni_2_P/FeP@NPC. Previous reports have proved that the incorporation of the N species in the catalysts could efficiently improve the intrinsic electrocatalytic activities, which could effectively improve the catalytic performance [28]. All of these results demonstrated that Ni_2_P/FeP@NPC catalysts with the combination of conductive N, P-doped carbon and highly active Ni_2_P/FeP heterostructures have been established.

**Figure 3:**
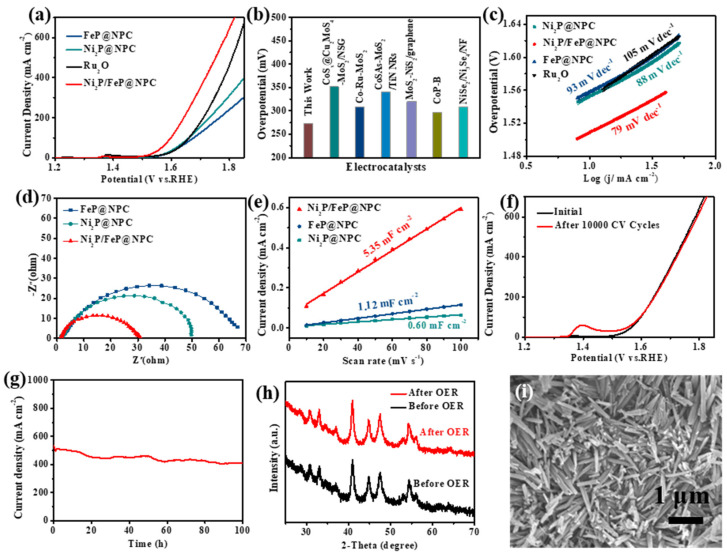
(**a**) The OER polarization curves of different catalysts in 1.0 M KOH solution; (**b**) Comparison of the overpotentials obtained at 10 mA cm^−2^ among Ni_2_P/FeP@NPC and other reported OER catalysts (references are provided in Table S1); (**c**) The relevant Tafel plots of different catalysts; (**d**) Nyquist plots of different catalysts; (**e**) Double-layer capacitance measurements to calculate the electrochemically active surface areas; (**f**) OER LSV curves of Ni_2_P/FeP@NPC before (black) and after (red) 10,000 CV cycles; (**g**) The chronopotentiometry curves of the FeP/Ni_2_P electrode after 100 h examination; (**h**) PXRD pattern, and (**i**) SEM image of Ni_2_P/FeP@NPC after chronoamperometric measurements for OER.

The catalytic performance of the Ni_2_P/FeP@NPC hybrid catalyst was then investigated. Firstly, the OER activity of the Ni_2_P/FeP@NPC catalysts in 1.0 M KOH electrolyte was investigated. As shown in Figure 3a, the representative polarization curves exhibited the geometric current density plotted against applied potential vs the reversible hydrogen electrode (RHE) of this Ni_2_P/FeP@NPC catalyst. The OER activities of Ni_2_P@NPC, FeP@NPC, and RuO_2_ were also evaluated by the same procedure. As shown in Figure 3a, Ni_2_P@NPC and FeP@NPC show larger overpotential of 621 and 699 mV at 400 mA cm^−2^, respectively, while Ni_2_P/FeP@NPC exhibited a much higher OER catalytic activity possessing the overpotential of 487 mV under the same condition. Specifically, the OER activities of other available bifunctional catalysts were compared with Ni_2_P/FeP@NPC in Figure 3b and Table S1. Experimental results exhibited that the catalyst in this work required the lowest overpotential of 273 mV to achieve 10 mA cm^−2^, indicating the potential application in overall water splitting at the small cell voltage. The OER kinetics of Ni_2_P/FeP@NPC was further investigated by the Tafel slope to disclose the inherent property of catalysts. As shown in Figure 3c, the measured Tafel slopes of Ni_2_P/FeP@NPC was 79 mV dec^−1^, was considerably lower than the counterparts of FeP (93 mV dec^−1^), RuO_2_ (105 mV dec^−1^), and Ni_2_P (88 mV dec^−1^). The much smaller Tafel slopes of Ni_2_P/FeP@NPC revealed the favorable OER kinetics of Ni_2_P/FeP@NPC. Electrochemical impedance spectroscopy (EIS) measurements of Ni_2_P@NPC, Ni_2_P/FeP@NPC and FeP@NPC in Figure 3d were conducted at OER conditions to reveal the charge transfer kinetics. The semicircle of Ni_2_P/FeP@NPC is much smaller than that of Ni_2_P@NPC and FeP@NPC, demonstrating that the bimetallic phosphide Ni_2_P/FeP@NPC possessed smaller charge transfer resistance. The electrochemically active surface area values of catalysts were further investigated by double-layer capacitances (Cdl), which were obtained by calculating the CV curves at different scan rates (Figures S5–S7). As shown in Figure 3e, the Cdl values of Ni_2_P/FeP@NPC (5.35 mF cm^−2^) are much higher than those of Ni_2_P (0.60 mF cm^−2^) and FeP (1.12 mF cm^−2^), demonstrating the higher exposed catalytic sites of Ni_2_P/FeP@NPC. Stability is another important indicator of electrocatalysts for practical applications. We further explored the stability of Ni_2_P/FeP@NPC by the long-term cycling test and amperometric i–t measurement. As illustrated in Figure 3f, the polarization curve of Ni_2_P/FeP@NPC after 10,000 CV cycles almost overlaps with the initial curve. Furthermore, the Ni_2_P/FeP@NPC maintained good stability for 100 h at 500 mA cm^−2^ (Figure 3g), which proves the long-term durability of Ni_2_P/FeP@NPC under large current density.

Because of the fantastic OER durability of Ni_2_P/FeP@NPC, we further tried to investigate the origin of OER performance. XRD, SEM and XPS were used to study the characteristics of Ni_2_P/FeP@NPC after OER stability testing (at the current density of 500 mA cm^−2^ for 100 h). As shown in Figure 3h, the XRD pattern of Ni_2_P/FeP@NPC did not change obviously after OER, implying that the crystal structure remained unchanged. Meanwhile, the morphology of the sample after OER was tested and presented in Figure 3i. It can be concluded that the Ni_2_P/FeP@NPC maintained its morphology and dimension after the stability testing, demonstrating the excellent corrosion resistance and chemical stability of Ni_2_P/FeP@NPC. XPS was further utilized to evaluate the change in the surface chemical composition and the electronic state after the catalytic process. The XPS spectrum after initial OER in Figure S8 exhibited the presence of Ni, Fe, C, P and N elements in the catalysts. The corresponding peaks of C, Fe, Ni, N and P in the high-resolution XPS spectrum exhibited little change with the appearance of the peak attributed to O = C-O (292.1 eV) (Figure S8b–f), suggesting the slight surface oxidation of Ni_2_P/FeP@NPC. The result agreed with XRD analysis, which proved that the N, P-doped carbon could effectively prevent the Ni_2_P/FeP from degradation, oxidation and corrosion during the OER. Therefore, the real active sites of Ni_2_P/FeP@NPC electrocatalyst for OER were the combination of N, P-doped carbon, and the Ni_2_P/FeP heterostructures.

**Figure 4:**
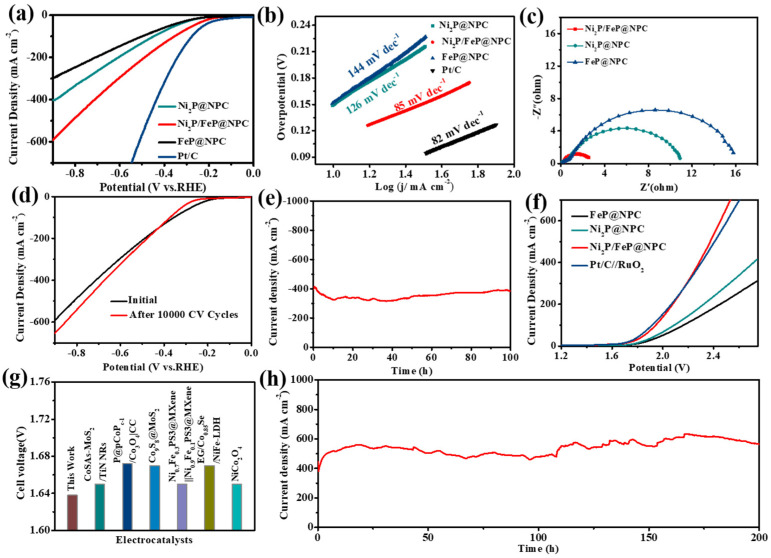
(**a**) The HER polarization curves of different catalysts; (**b**) The relevant Tafel plots of different catalysts; (**c**) Nyquist plots of different catalysts; (**d**) HER LSV curves of Ni_2_P/FeP@NPC before (black) and after (red) 10,000 CV cycles; (**e**) The chronopotentiometry curve of the Ni_2_P/FeP@NPC electrode tested for 100 h. (**f**) The polarization curves of different catalysts for overall water splitting. (**g**) Comparison of the potentials required at 10 mA cm^−2^ among Ni_2_P/FeP@NPC and the available reported catalysts for overall water splitting (References are provided in Table S2); (**h**) The chronopotentiometric curve of the Ni_2_P/FeP@NPC electrode tested at the constant current density of 500 mA cm^−2^ for 200 h.

Next, we further explored the possibility of Ni_2_P/FeP@NPC as a HER catalyst. As shown in Figure 4a, Ni_2_P/FeP@NPC exhibited considerable HER catalytic activity with low overpotentials of 182 mV to obtain the current density of 10 mA cm^−2^, while the much higher overpotentials of 217 and 214 mV were needed to achieve the current density of 10 mA cm^−2^ for FeP@NPC and Ni_2_P@NPC, respectively. Moreover, the calculated Tafel slope of Ni_2_P/FeP@NPC in Figure 4b was 85 mV dec^−1^, which was smaller than those of Ni_2_P (126 mV dec^−1^) and FeP (144 mV dec^−1^), indicating that Ni_2_P/FeP@NPC possessed the fastest HER kinetic process. Moreover, EIS was also conducted to evaluate the intrinsic HER electrocatalytic kinetics. The corresponding Rct of Ni_2_P/FeP@NPC was smaller than those of Ni_2_P@NPC and FeP@NPC, demonstrating the higher transfer coefficient and electronic conductivity (Figure 4c). Furthermore, the Ni_2_P/FeP@NPC catalyst possessed excellent stability during long-time HER operations. As shown in Figure 4d, the catalytic performance exhibited no apparent deterioration after 10,000 cycles. As shown in Figure 4e, Ni_2_P/FeP@NPC maintained the current density of 400 mA cm^−2^ for 100 h without obvious decline, demonstrating the fantastic durability of Ni_2_P/FeP@NPC in the HER process. Such excellent HER durability of Ni_2_P/FeP@NPC ranks at the top level of the reported HER electrocatalysts.

Because of the fantastic catalytic activity of Ni_2_P/FeP@NPC for HER and OER, the two-electrode water splitting device was constructed using Ni_2_P/FeP@NPC as the bifunctional catalyst for HER and OER. Remarkably, the cell voltage to afford the current density of 10 mA cm^−2^ was low to 1.64 V (Figure 4f), which was comparable to the coupled benchmark RuO_2_//Pt/C catalysts (1.63 V), and better than many previously reported bifunctional electrocatalysts (Figure 4g and Table S2). In addition, as shown in Figure 4h, the device exhibited outstanding stability for 200 h after the long-term test at the current density of 500 mA cm^−2^.

**Figure 5:**
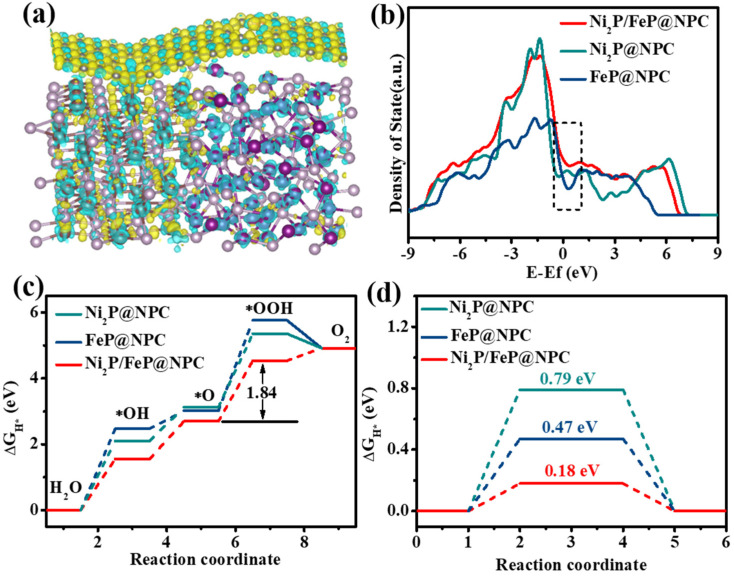
(**a**) Differential charge density between N, P-doped carbon and Ni_2_P/FeP in the heterostructure (gray: C, purple: Ni, brown: Fe, pink: P, orange: N, white: H); (**b**) The density of states (DOS) of Ni_2_P@NPC, FeP@NPC, and Ni_2_P/FeP@NPC; (**c**) The standard free energy diagrams in the OER process of Ni_2_P@NPC, FeP@NPC, and Ni_2_P/FeP@NPC; (**d**) Gibbs free energy (DG) profile of the HER on Ni_2_P@NPC, FeP@NPC, and Ni_2_P/FeP@NPC.

To clarify the interfacial charge properties, theoretical models of Ni_2_P@NPC, FeP@NPC, and Ni_2_P/FeP@NPC heterostructures were established (Figure S9) and DFT calculations were utilized to evaluate their surface energetics. Figure 5a shows the differential charge density between N, P-doped carbon and Ni_2_P/FeP in the heterostructure. Yellow and cyan refer to the positive and negative charges, respectively. As shown in Figure 5a, the differential charge density at the Ni_2_P/FeP@NPC interface indicated that the significant electron redistribution was achieved through the extensive electron transformation from the Ni_2_P/FeP side to the NPC region. Thus, the NPC side was electron accumulating, whereas the Ni_2_P/FeP domain was electron depleting, which indicated the strong electronic interaction between these two domains. This kind of interfacial electronic structure was beneficial for the surface adsorption of the intermediate. As shown in Figure 5b, the density of states (DOS) was further conducted to evaluate their electrical conductivity. The results showed that the Ni_2_P/FeP@NPC possessed higher carrier density around the Fermi level compared with Ni_2_P@NPC and FeP@NPC, which demonstrated that better OER catalytic activity could be achieved through the improvement of the electrical conductivity. The intrinsic OER catalytic activities were then analyzed by calculating the surfaces Gibbs free energy (Δ*G*) profiles of Ni_2_P@NPC, FeP@NPC, and Ni_2_P/FeP@NPC models through a four-step pathway at alkaline conditions (Figure S10):

* + OH^−^ →^*^OH + e^−^ (1)

*OH + OH^−^ → H_2_O + *O + e^−^ (2)

*O + OH^−^ →*OOH + e^−^ (3)

*OOH + OH^−^ →*+ O_2_ + e^−^ (4)

where * denotes the active sites on the catalyst surface. As shown in Figure 5a, the Δ*G* diagrams demonstrated that all electrocatalysts possessed the same rate-determining steps (RDS), which was the formation of the *OOH intermediate in the third step. The Ni_2_P/FeP@NPC electrocatalyst displayed the smallest total energy barrier (Δ*G*: 1.84 eV) compared with the Ni_2_P@NPC (2.23 eV) and FeP@NPC (2.75 eV). These results indicated that the formation of the heterointerface could optimize the adsorption and desorption steps in Ni_2_P/FeP@NPC to accelerate the whole OER process. As for the HER process, the intrinsic HER activities of the catalysts could be investigated through the Gibbs free energy of hydrogen (ΔG_H*_) adsorption. The negative ΔG_H*_ is beneficial for the adsorption of H* but generates side effects on the desorption of products while too positive ΔG_H*_ does the opposite. When ΔG_H*_ is tending to zero, the H* and H_2_ are more easily adsorbed and desorbed at the active center, which could be beneficial for the HER. The Ni_2_P/FeP@NPC presented the positive ΔG_H*_ of 0.18 eV (Figure 5d), which was much lower than Ni_2_P@NPC (0.79 eV) and FeP@NPC (0.47 eV), confirming that the interaction between Ni_2_P/FeP and N, P-doped carbon could adjust the ΔG_H*_ to zero to facilitate the reaction.

All above theoretical/experimental experiments demonstrated that the fantastic catalytic activities of Ni_2_P/FeP@NPC were ascribed to the three-phase heterojunction interface constructed by NPC, Ni_2_P and FeP, which could effectively optimize the desorption of intermediates and thus significantly boost the kinetics in OER, HER, and the overall water splitting process. The heterojunction interface optimized the adsorption/desorption ability of intermediates (O*, OH*, OOH* and H*), and the inner Ni_2_P/FeP possess superior electrical conductivity and high electron density near the Fermi level, which has better conductivity and a high transfer coefficient, could produce synergistic effects in promoting both HER and OER performance.

## 3. Materials and Methods

### 3.1. Synthesis of MOF Precursors

Typically, STA-12 was synthesized by hydrothermal as early reported. [24] In brief, N, N’-piperazinebis(methylenephosphonic acid) was synthesized using a variation of the modified Mannich reaction of piperazine, using HCl as a catalyst. In a typical synthetic process of Ni-STA-12, nickel acetate tetrahydrate (0.369 g, 0.185 mmol) was stirred together with N, N’-Piperazinebis(methylenephosphonic acid) (0.263 g, 0.93 mmol) in 15 mL deionized water. The pH of the solution was adjusted to 8 by the addition of potassium hydroxide solution. The acidified solution was then placed in a 25 cm^3^ Teflon-lined autoclave and heated at 160 °C for 48 h. The NiFe-STA-12 or Fe-STA-12 contrasts were synthesized by adjusting the corresponding metal salt, while the other experimental processes and parameters were kept unchanged. The resultant crystalline materials were thoroughly washed with deionized water several times and dried at 80 °C for 12 h under vacuum.

### 3.2. Synthesis of the Ni_2_P@NPC, Ni_2_P/FeP@NPC and FeP@NPC

In a typical phosphorization procedure, 500 mg sodium hypophosphite powder was positioned upstream of the tube as the phosphorous source. Subsequently, the as-obtained MOF precursor (100 mg) was positioned at the center of the tube and phosphorized under argon vapor at 450 °C for 2 h. After cooling, the obtained product was denoted Ni_2_P@NPC, Ni_2_P/FeP@NPC and FeP@NPC, respectively.

## 4. Conclusions

In summary, the N, P-doped carbon layer-encapsulated Ni_2_P/FeP heterojunction nanorods (Ni_2_P/FeP@NPC) were successfully engineered and synthesized. As revealed by the DFT results, the electrons could redistribute among the N, P-doped carbon, Ni_2_P, and FeP, to optimize the adsorption energy of H- and O-containing intermediates, which endows the best ΔG_H*_ for HER and decreases the ΔG of RDS for OER to improve the HER/OER intrinsic activity. The Ni_2_P/FeP@NPC exhibited excellent activity for HER (ƞ_10_ = 182 mV) and OER (ƞ_10_ = 273 mV), which was much better than Ni_2_P@NPC (HER: ƞ_10_ = 214 mV, OER: ƞ_10_ = 323 mV) and FeP@NPC (HER: ƞ_10_ = 217 mV, OER: ƞ_10_ = 330 mV). Meanwhile, the heterojunction could effectively facilitate electronic transfers at large current density and the carbon layers could avoid metal dissolution in harsh solutions, which makes Ni_2_P/FeP@NPC exhibit excellent stability for water splitting. This work provides fantastic catalytic candidates for industrial electrochemical water splitting.

## Data Availability

The data presented are available from the authors.

## Supplementary Materials

The following supporting information can be downloaded at: https://www.mdpi.com/article/10.3390/molecules28052280/s1, Figure S1: Raman patterns of Ni_2_P@NPC, Ni_2_P/FeP@NPC and FeP@NPC; Figure S2: (a) SEM images of Ni_2_P@NPC and (b) FeP@NPC; Figure S3: (a) TEM image of Ni_2_P/FeP@NPC and the corresponding EDX element mapping for (b) C, (c) Ni, (d) Fe, (e) P and (f) N; Figure S4: Survey scan of Ni_2_P/FeP@NPC; Figure S5: Cyclic voltammograms for Ni_2_P@NPC with different scan rates from 10 to 100 mV s^−1^ in the potential range of 1.1–1.30 V versus RHE; Figure S6: Cyclic voltammograms for FeP@NPC with different scan rates from 10 to 100 mV s^−1^ in the potential range of 1.1–1.30 V versus RHE; Figure S7: Cyclic voltammograms for Ni_2_P/FeP@NPC with different scan rates from 10 to 100 mV s^−1^ in the potential range of 1.1–1.30 V versus RHE; Figure S8: (a) Survey scan of Ni_2_P/FeP@NPC; High-resolution XPS spectra of (b) C, (c) Fe (d) Ni, (e) N and (f) P in Ni_2_P/FeP@NPC; Table S1: Comparison of the OER activities with available robust catalysts; Table S2: Comparison of the overall water splitting activities with available robust bifunctional catalysts; Figure S9: Atomic models of (a) Ni_2_P@NPC, (b) FeP@NPC and (c) Ni_2_P/FeP@NPC (gray: C, purple: Ni, brown: Fe, pink: P, orange: N, white: H); Figure S10: Atomic models of (a), (b) and (c) Ni_2_P@NPC, (d), (e) and (f) FeP@NPC and (g), (h) and (i) Ni_2_P/FeP@NPC absorbed OH, O and OOH intermediates (gray: C, purple: Ni, brown: Fe, pink: P, orange: N, white: H, red: O).
