# Supplementary material for: Metal–Organic Framework Derived Ni2P/FeP@NPC Heterojunction as Stability Bifunctional Electrocatalysts for Large Current Density Water Splitting
Source: Molecules. 2023 Feb 28;28(5):2280. doi: 10.3390/molecules28052280 (PMC10005255; doi:10.3390/molecules28052280)
Supplement: Supplementary file 1 [file molecules-28-02280-s001.zip › molecules-2187932-supplementary.pdf]

Supporting Information

Metal–organic framework derived $Ni_2P/FeP@NPC$ heterojunction as stability bifunctional electrocatalysts for large current density water splitting

Huimin Jiang[1], *Shuo Zhang*[1], *Qiuju Fu*[1], *Liting Yan*[2], *Jun Zhang*[1]*, *Xuebo Zhao*[1,2]*

[1] College of Chemical Engineering, China University of Petroleum (East China), Qingdao, 266580, China

[2] School of Materials Science and Engineering, Qilu University of Technology (Shandong Academy of Sciences), 250353, Jinan, China

* Corresponding author.
E-mails: zhangj@upc.edu.cn (J.Z.); .
zhaoxuebo@upc.edu.cn (X. Zhao)

1. Characterization

Powder X-ray diffraction (XRD) data were obtained on an XRD diffractometer (Rigaku, Japan) with Cu Ka radiation. The SEM images were acquired using a Merlin microscope (Carl Zeiss, Germany). The TEM images were obtained using a JEM2100F transmission electron microscope (JEOL Co. Ltd, Japan). Nitrogen sorption isotherms were measured at 77 K using an Autosorb volumetric gas sorption analyzer (Quantachrome, USA). All of the samples were degassed in vacuum for 12 h at 100 ℃ to fully remove guest molecules prior to analysis.

2. Chemicals

Piperazine ($C_4H_{10}N_2$, 95%), phosphorous acid (AR, 99%) were purchased from Shanghai Macklin Biochemical Co., Ltd. Hydrochloric acid (HCl, 37%) was purchased from Sinopharm Chemistry. Iron(II) acetate ($C_4H_6O_4Fe$, 98%) and nickel acetate ($Ni(CH_3COO)_2$, 98%) were purchased from Energy Chemical.

3. Electrochemical experiments

Electrochemical measurements were performed on a CHI 760E electrochemical workstation (CH Instrument, Chenhua Co., Shanghai, China). A conventional three-electrode system was adopted, a graphite rod as the counter electrode, and a mercuric oxide electrode (Hg/HgO) electrode as the reference electrode. Herein, all of the potentials were normalized to the reversible hydrogen electrode (RHE).

For typically preparing the catalyst ink, 10 mg of the catalyst was put into the mixture containing 0.45 mL of ethanol, 0.45 mL of water and 100 μL of Nafion (5 wt%). Next, the mixture was further ultrasonicated for at least 30 min to obtain a homogeneous suspension. After that, the as-prepared ink was drop casted onto nickel foam to give a catalyst loading of 0.2 mg cm^{-2}. In the controls, commercial Pt/C and RuO_2 catalysts modified electrodes were constructed in a similar way with the mass loading of 0.2 mg cm^{-2}.

4. Computational methods

Density functional theory (DFT) calculations were implemented in the Vienna ab initio simulation software package (VASP) and performed by applying the plane-wave pseudopotential technique.[1] The electron exchange-correlation energy was simulated by the Perdew-Burke-Ernzerhof (PBE) functional within the generalized gradient approximation (GGA).[2] Utilizing the projection augmented wave (PAW) pseudopotentials to describe the ion-electron interactions.[3] All the structures were relaxed using the conjugate gradient method until the force component on each atom was less than 0.02 eV/Å, and the total energy convergence criterion of the self-consistent field method was set to 10^{-5} eV.

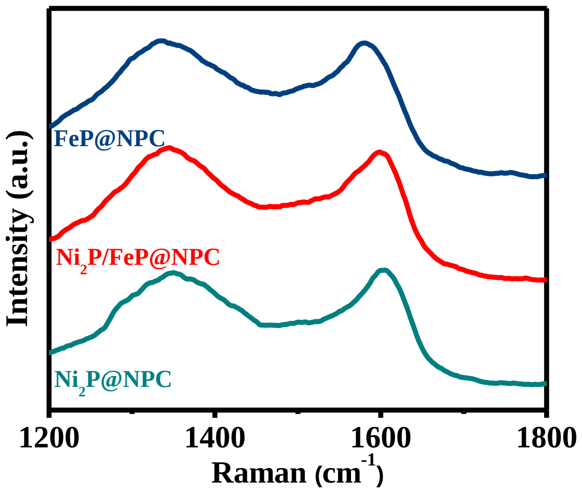

Figure S1. Raman patterns of Ni_2P@NPC, Ni_2P/FeP@NPC and FeP@NPC.

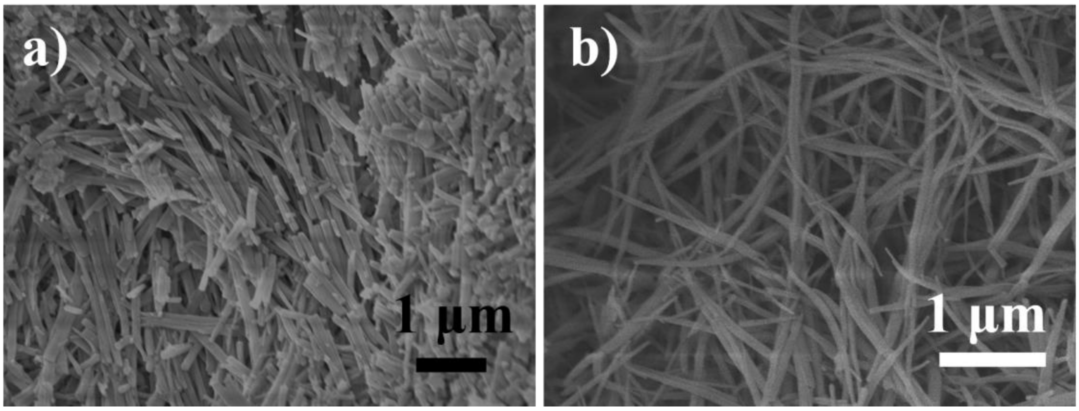

Figure S2. (a) SEM images of Ni_2P@NPC and (b) FeP@NPC.

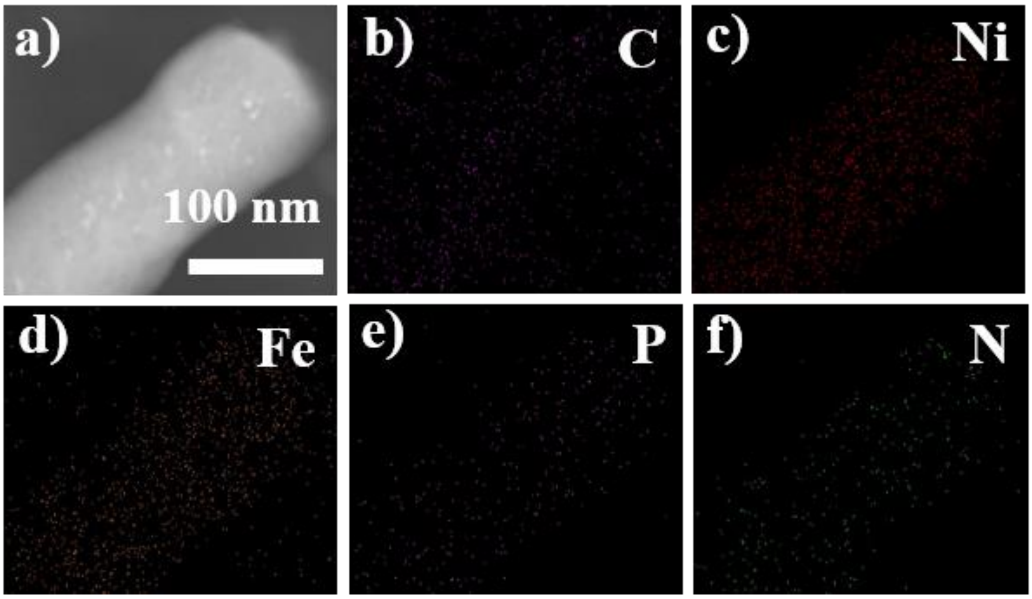

Figure S3. a) TEM image of Ni_2P/FeP@NPC and the corresponding EDX element mapping for b) C, c) Ni, d) Fe, e) P and f) N.

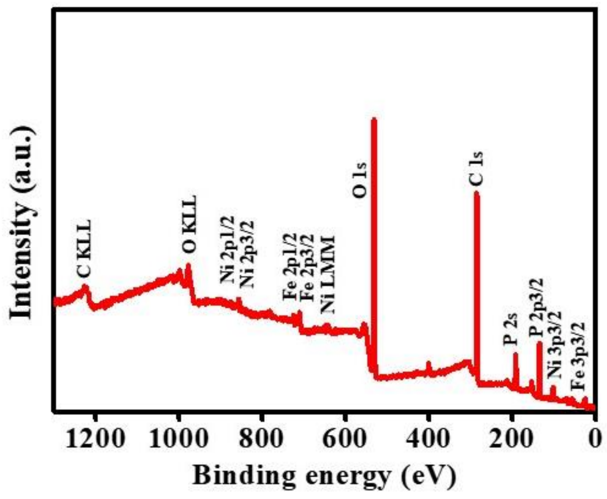

Figure S4. Survey scan of Ni_2P/FeP@NPC.

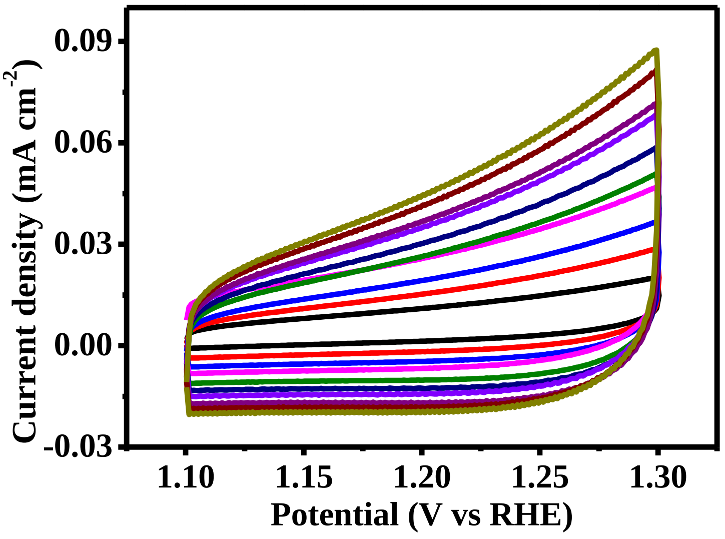

Figure S5. Cyclic voltammograms for Ni_2P@NPC with different scan rates from 10 to 100 mV s^{-1} in the potential range of 1.1-1.30 V versus RHE.

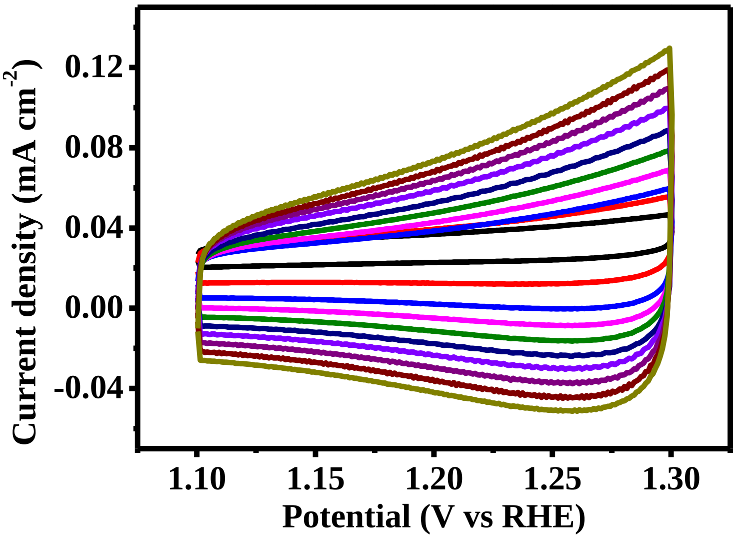

Figure S6. Cyclic voltammograms for FeP@NPC with different scan rates from 10 to 100 mV s^{-1} in the potential range of 1.1-1.30 V versus RHE.

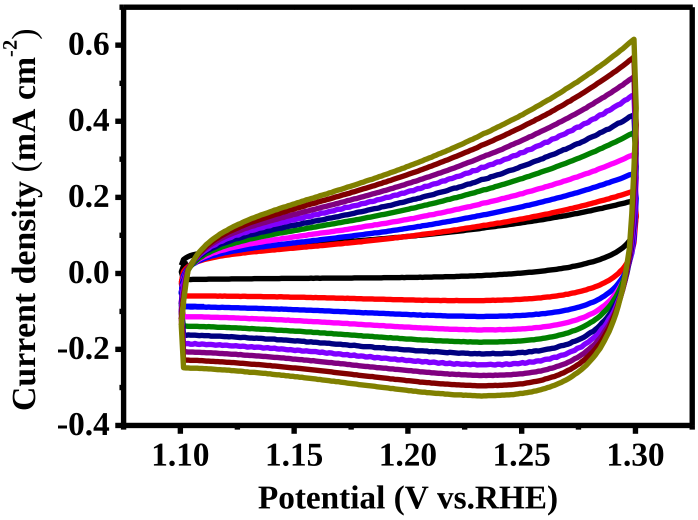

Figure S7. Cyclic voltammograms for Ni_2P/FeP@NPC with different scan rates from 10 to 100 mV s^{-1} in the potential range of 1.1-1.30 V versus RHE.

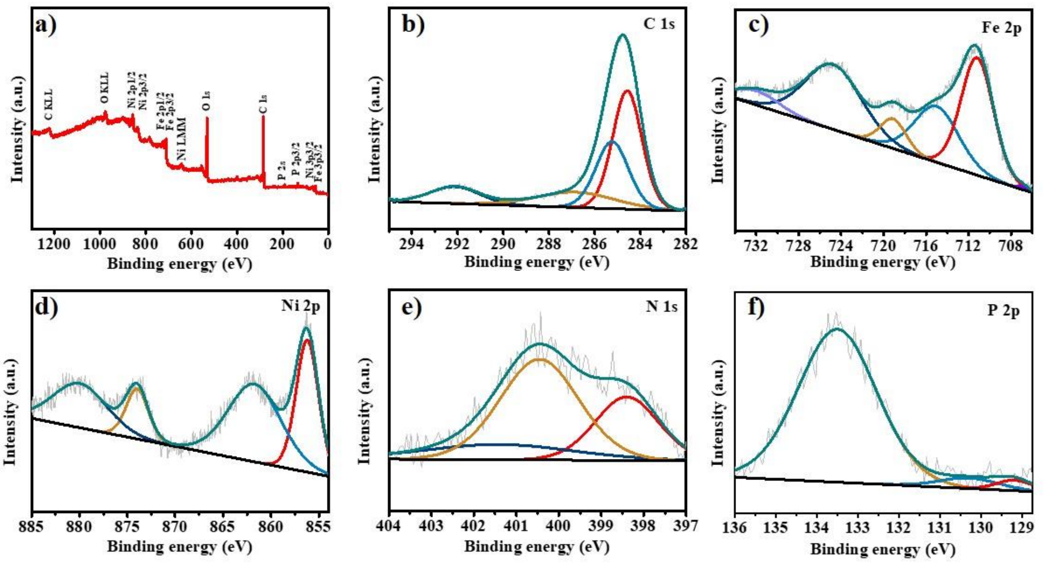

Figure S8. (a) Survey scan of Ni_2P/FeP@NPC; High-resolution XPS spectra of b) C, c) Fe d) Ni, e) N and f) P in Ni_2P/FeP@NPC.

Table S1. Comparison of the OER activities with available robust catalysts.

OER catalysts	Electrolytes	η_{10}, OER (mV)	References
MoS_2-NiS_2/graphene	1.0 M KOH	320	4
CoS_x@Cu_2MoS_4-MoS_2/NSG	1.0 M KOH	351.4	5
Co-Ru-MoS_2	1.0 M KOH	308	6
CoSAs-MoS_2/TiN NRs	1.0 M KOH	340.6	7
P@pCoP$_{c-1}$/Co_3O_4|CC	1.0 M KOH	320	8
CoP-B	1.0 M KOH	297	9
$NiSe_2$/Ni_3Se_4/NF	1.0 M KOH	309	10
Ni_2P/FeP@NPC	1.0 M KOH	273	This work

Table S2. Comparison of the overall water splitting activities with available robust bifunctional catalysts.

Catalysts	Electrolytes	Voltage at η_{10} (V)	References
Co_9S_8@MoS_2 couple	1.0 M KOH	1.67	11
CoSAs-MoS_2/TiN NRs	1.0 M KOH	1.65	7
P@pCoP$_{c-1}$/Co_3O_4|CC	1.0 M KOH	1.672	8
$Ni_{0.7}Fe_{0.3}PS_3$@MXene|| $Ni_{0.9}Fe_{0.1}PS_3$@MXene couple	1.0 M KOH	1.65	9
$NiCo_2O_4$	1.0 M KOH	1.65	13
EG/$Co_{0.85}$Se/NiFe-LDH	1.0 M KOH	1.67	14
Ni_2P/FeP@NPC	1.0 M KOH	1.64	This work

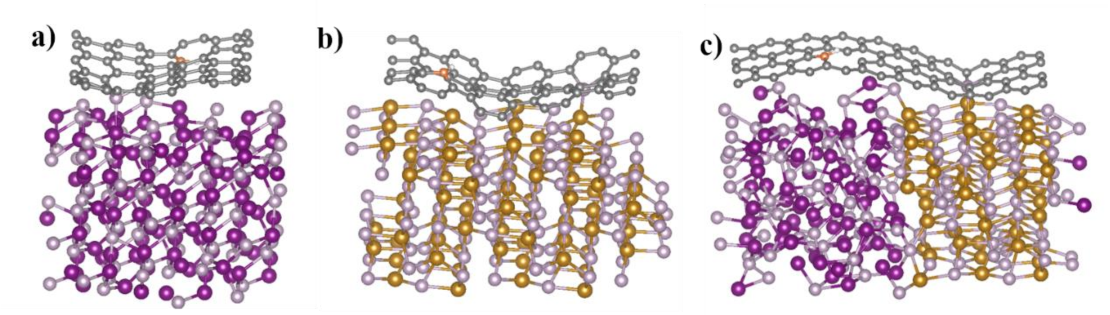

Figure S9. Atomic models of a) Ni_2P@NPC, b) FeP@NPC and C) Ni_2P/FeP@NPC (gray: C, purple: Ni, brown: Fe, pink: P, orange: N, white: H).

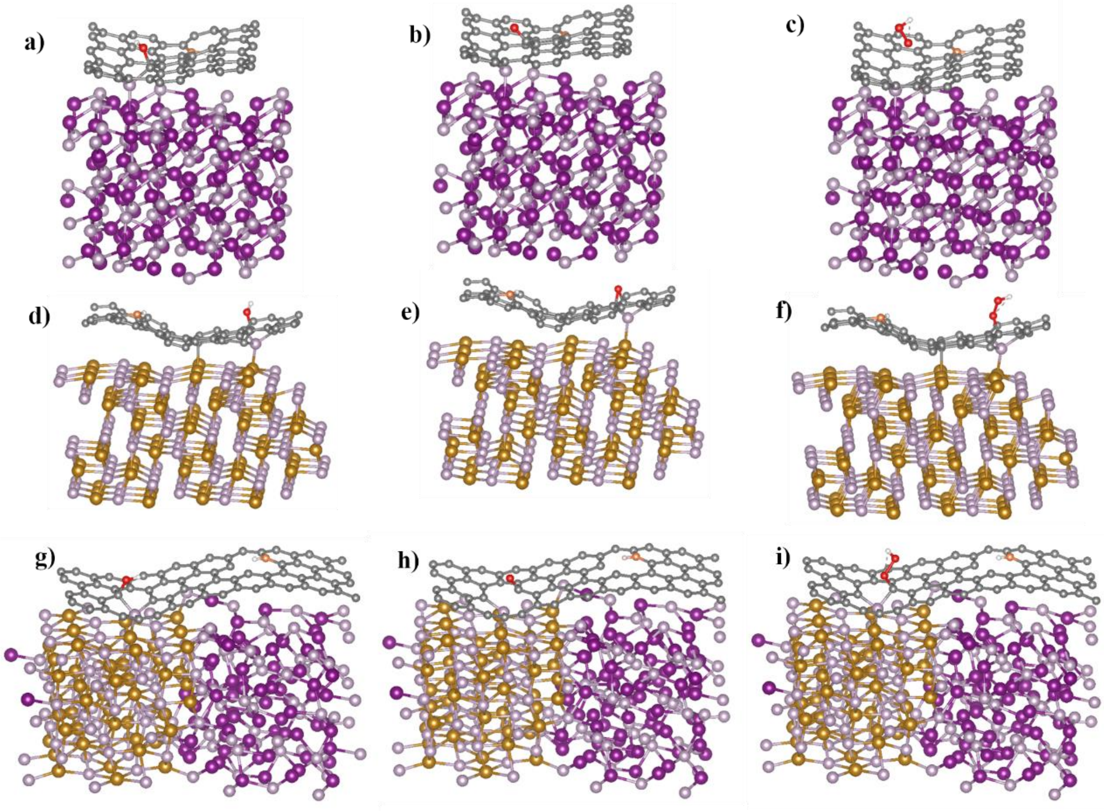

Figure S10. Atomic models of a), b) and c) Ni_2P@NPC, d), e) and f) FeP@NPC and g), h) and i) Ni_2P/FeP@NPC absorbed OH, O and OOH intermediates (gray: C, purple: Ni, brown: Fe, pink: P, orange: N, white: H, red: O).

References

[1] G. Kresse, J. Furthmüller, *Phys. Rev. B* **1996**, 54, 11169.

[2] J. P. Perdew, K. Burke, M. Ernzerhof, *Phys. Rev. Lett.* **1996**, 77, 3865.

[3] P. Blöchl, *Phys. Rev. B* **1994**, 50, 17953.

[4] S. P. Lonkar, V. V. Pillai, S. M. Alhassan, *Mater. Adv.* **2020**, 1, 794.

[5] D. C. Nguyen, D. T. Tran, T. L. L. Doan, D. H. Kim, N. H. Kim, J. H. Lee, Adv. Energy Mater. 2020, 10, 1903289.

[6] I. S. Kwon, T. T. Debela, I. H. Kwak, Y. C. Park, J. Seo, J. Y. Shim, S. J. Yoo, J. G. Kim, J. Park, H. S. Kang, *Small* **2020**, 16, 2000081.

[7] T. L. L. Doan, D. C. Nguyen, S. Prabhakaran, D. H. Kim, D. T. Tran, N. H. Kim, J. H. Lee, *Adv. Funct. Mater.* **2021**, 31, 2100233.

[8] Y. Kim, D. Kim, J. Lee, L. Y. S. Lee, D. K. Ng, *Adv. Funct. Mater.* **2021**, 31, 2103290.

[9] G. Yuan, J. Bai, L. Zhang, X. Chen, L. Ren, *Appl. Catal., B* **2021**, 284, 119693.

[10] L. Tan, J. Yu, H. Wang, H. Gao, X. Liu, L. Wang, X. She, T. Zhan, Appl. Catal. B: Environ. **2022**, 303, 120915.

[11] J. Bai, T. Meng, D. Guo, S. Wang, B. Mao, M. Cao, *ACS Appl. Mater. Interfaces* **2018**, 10, 1678.

[12] C. F. Du, K. N. Dinh, Q. Liang, Y. Zheng, Y. Luo, J. Zhang, Q. Yan, *Adv. Energy Mater.* **2018**, 8, 1801127.

[13] X. Gao, H. Zhang, Q Li, X Yu, Z. Hong, X Zhang, *Angew. Chem. Int. Ed.* **2016**, 55, 6290-6294.

[14] Y. Hou, M. R. Lohe, J. Zhang, S. Liu, X. Zhuang, X. Feng, *Energy Environ. Sci.* **2018**, 9, 478-483.
